# Aging and sex: Impact on microglia phagocytosis

**DOI:** 10.1111/acel.13182

**Published:** 2020-07-29

**Authors:** Natalia Yanguas‐Casás, Andrea Crespo‐Castrillo, Maria‐Angeles Arevalo, Luis Miguel Garcia‐Segura

**Affiliations:** ^1^ Consejo Superior de Investigaciones Científicas (CSIC) Instituto Cajal Madrid Spain; ^2^ Centro de Investigación Biomédica en Red de Fragilidad y Envejecimiento Saludable (CIBERFES) Instituto de Salud Carlos III Madrid Spain; ^3^ Present address: IIS Puerta de Hierro‐Segovia de Arana (IDIPHISA) Majadahonda Spain

**Keywords:** aging, dysfunction, irresponsiveness, Microglia, neuroinflammation, phagocytosis, sex differences

## Abstract

Microglia dysfunction and activation are important hallmarks of the aging brain and are concomitant with age‐related neurodegeneration and cognitive decline. Age‐associated changes in microglia migration and phagocytic capacity result in maladaptive responses, chronic neuroinflammation, and worsened outcomes in neurodegenerative disorders. Given the sex bias in the incidence, prevalence, and therapy response of most neurological disorders, we have here examined whether the phagocytic activity of aged microglia is different in males and females. With this aim, the phagocytosis activity of male and female cells was compared in an in vitro aged microglia model and in microglia isolated from adult (5‐month‐old) or aged (18‐month‐old) mice. In both models, the phagocytosis of neural debris increased with aging in male and female cells and was higher in aged female microglia than in aged male cells. However, female aged microglia lost its ability to adapt its phagocytic activity to inflammatory conditions. These findings suggest that microglia phagocytosis of neural debris may represent a previously unexplored neuroprotective characteristic of aged microglia that may contribute to the generation of sex differences in the manifestation of neurodegenerative diseases.

## Graphical abstract

Sex differences in postnatal microglia: higher pathogen‐specific phagocytosis is detected in male cells while females show an enhanced nonspecific and neural debris phagocytosis compared to the other sex. Phagocytosis of neural debris increased with aging in male and female cells. These characteristics of the phagocytic behaviour of microglia are potential contributors to the generation of sex differences in the manifestation of neurodegenerative diseases.

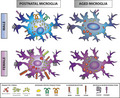

## 1 INTRODUCTION

Microglia are the primary innate immune cells of the brain and are key players in the resolution or propagation of the inflammatory process (Kettenmann, Hanisch, Noda, & Verkhratsky, 2011; Ransohoff & Perry, 2009). These glial cells perform central functions in the regulation of cell number, synaptic patterning, homeostasis maintenance, and response to pathogens by selective phagocytosis. Microglia phagocytosis is a fine‐tuned process mediated by the expression of specific receptors on the cell surface and downstream signaling pathways that, depending on their ability to recognize misfolded proteins and apoptotic cells or bind structurally conserved molecules derived from microbial pathogens (such as Toll‐like receptors; TLRs), contribute to the engulfment of cellular debris, harmful particles, or synapses (Fu, Shen, Xu, Luo, & Tang, 2014; Galloway, Phillips, Owen, & Moore, 2019).

The incidence of most neurological disorders increases with aging, and two of its common characteristics, chronic neuroinflammation and impairment of microglial responses (Bachiller et al., 2018), are affected by the aging process. Indeed, microglia become senescent/dystrophic and less responsive to stimulation with age, and the mechanisms underlying age‐dependent phenotypic changes vary from extrinsic environmental changes to intrinsic changes in genomic integrity (Rawji et al., 2016; Streit & Xue, 2013). The aged microglial phenotype is characterized by reduced migration and phagocytosis activity, as well as by exacerbated inflammatory responses (microglia priming) and deficits in chemotactic functions (Damani et al., 2011; Koellhoffer, McCullough, & Ritzel, 2017). Overall, age‐related alterations in the expression of receptors implicated in innate immunity and phagocytosis, and the inability to mount a normal response to injury or inflammation, limit the capacity of microglia to cope with pathogens or neurodegeneration and contribute to an increased susceptibility and neurodegeneration (Liang, Domon, Hosur, Wang, & Hajishengallis, 2009).

Although there are robust sex differences in the epidemiology, clinical features, and pathophysiology of many neurological disorders (The Lancet, 2019), little attention has been paid to the sex differences in microglia function with aging. In this study, we aimed to determine the relevance of sex in the aged phenotype of microglia, analyzing two key functional responses of these cells: migration and phagocytosis. With this aim, we studied microglia isolated from adult or aged mouse brains (from 5‐ and 18‐month‐old animals, respectively) and in an experimental aging model in vitro after isolation of microglia from neonatal mouse brains.

## 2 RESULTS

### 2.1 Microglia phagocytosis is affected by aging in a sex‐specific way

**Figure 1:**
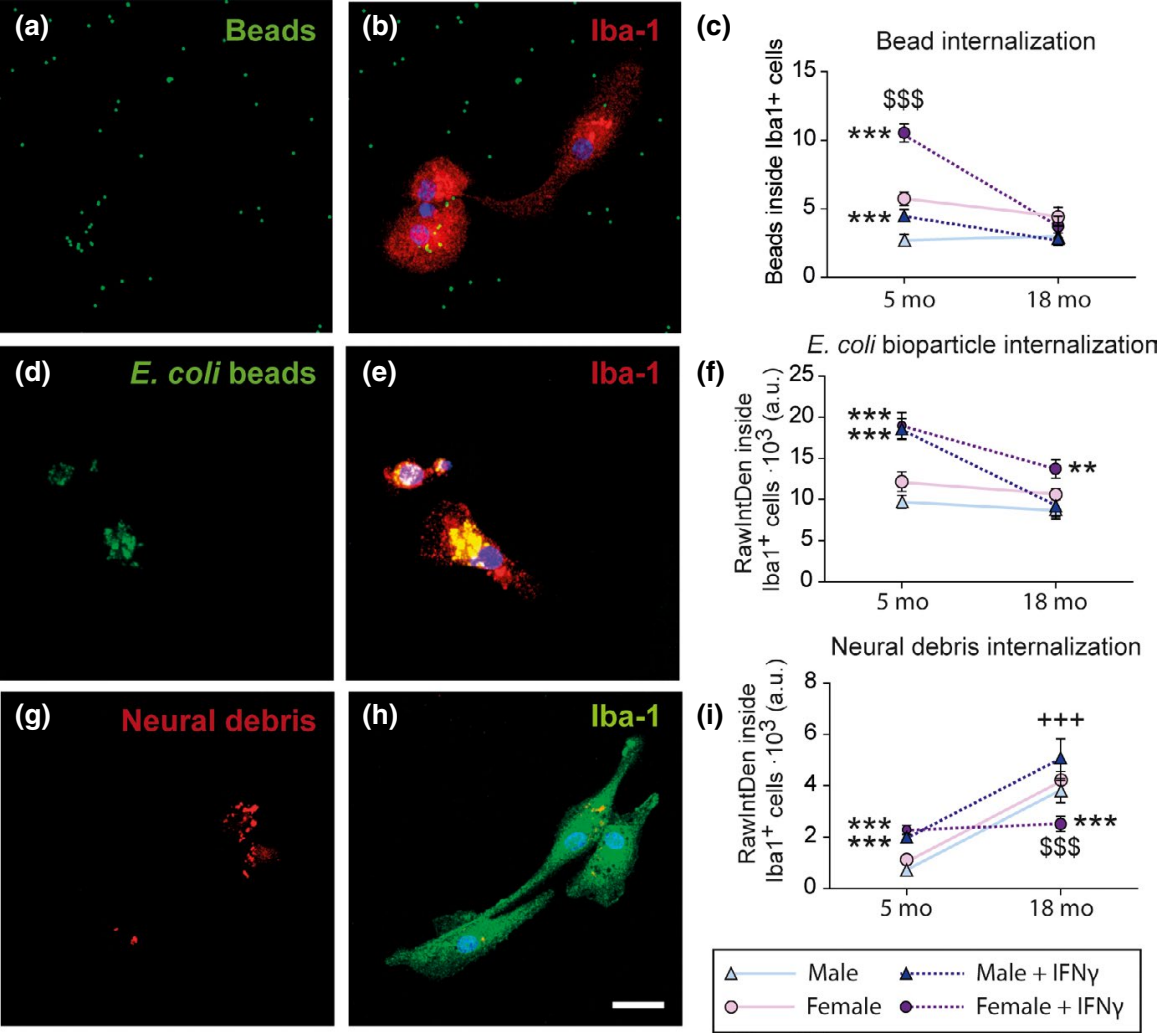
Phagocytosis of microglia purified from adult (5 months) or aged (18 months) mouse brain. Representative images of microglia: nonspecific bead intake (a, b), pathogen‐specific (d, e), and neural debris (g, h) phagocytosis. (c, f, i) Amount of internalized particles per cell. ^+++^ *p* < .001 effect of time measured by two‐way ANOVA followed by Bonferroni post hoc test; ^$$$^ *p* < .001 sex differences, **p < .01, ***p < .001 effect of IFN‐γ treatment measured by one‐way ANOVA followed by Tukey's post hoc test. Dots show mean ± *SEM*. Blue: male; Purple: female; Dark: IFN‐γ treatment

As previously mentioned, microglia migration, motility (Damani et al., 2011; Hefendehl et al., 2014), and phagocytic activity are impaired with aging (Damani et al., 2011; Koellhoffer et al., 2017). Some studies had found sex differences in the phagocytic activity of microglia in early developmental stages or even in adulthood (Hanamsagar & Bilbo, 2016; Villa et al., 2018; Yanguas‐Casás et al., 2018); however, possible sex differences in microglia phagocytosis had not been assessed in aged brains. Therefore, we decided to focus our studies on the phagocytic responses of microglial cells directly purified from adult and aged mouse brains. For this, we performed three different engulfment assays to evaluate: (a) nonspecific phagocytosis (quantifying fluorescent bead intake); (b) pathogen‐specific phagocytosis (measuring *Escherichia coli* bioparticles uptake); and (c) neural debris phagocytosis (analyzing the intake of Cy^TM^3‐labeled neural debris) (Figure 1), using IFN‐γ as a pro‐phagocytic stimulus (Yanguas‐Casás et al., 2018). Then, we measured the amount of internalized particles in actively engulfing microglial cells in the different experimental conditions.

Male and female microglia purified from adult (5 months) brains showed similar internalization of uncoated beads (nonspecific phagocytosis), *E. coli* bioparticles (pathogen‐specific phagocytosis), and neural debris under basal conditions (Figure 1). The comparison of the results obtained with microglia purified from adult and aged brains revealed that aging had significant effects on basal microglia phagocytosis of neural debris. Thus, both male and female microglia isolated from aged brain (18 months) significantly increased the internalization of neural debris compared to microglia isolated from adult brains (Figure 1i).

IFN‐γ treatment increased nonspecific, pathogen‐specific, and neural debris intake in both male and female microglia purified from adult brains (Figure 1c,f,i). However, adult female microglia showed a much higher increase in bead internalization than adult male microglia (Figure 1c). This sex difference was lost in microglia isolated from aged brains, in which bead phagocytosis was irresponsive to IFN‐γ stimulation in both sexes (Figure 1c). In addition, aged male microglia did not increase the internalization of *E. coli* bioparticles upon IFN‐γ stimulation (Figure 1f). Furthermore, IFN‐γ stimulation was ineffective to increase neural debris internalization in microglia isolated from aged female brains. Thus, aging affects microglia phagocytosis in response to an inflammatory challenge in a sex‐specific way.

### 2.2 Perinatal male and female microglia acquire a senescent‐like phenotype after 16 days in vitro

Previous studies had described an experimental model to reproduce irresponsive/senescent microglia in vitro (Caldeira et al., 2014); however, they did not evaluate the relevance of sex in the senescence process. For this reason, we characterized the senescent phenotype in microglia obtained separately from male and female mouse brains in this in vitro model.

**Figure 2:**
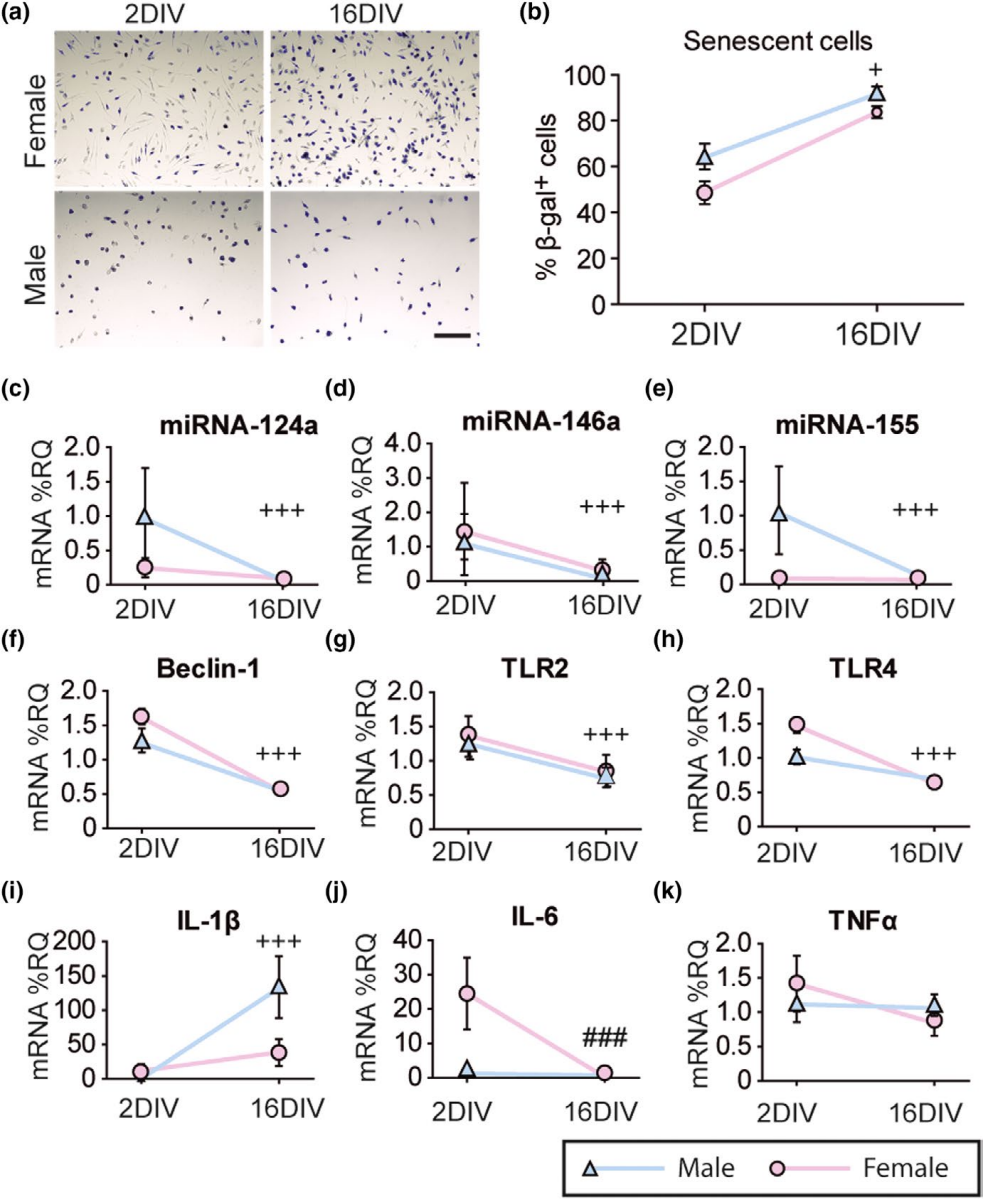
Acquisition of senescent‐like phenotype by male and female microglia at 16 days in vitro (DIV). (a) Representative images of β‐galactosidase activity (β‐gal, senescent cells, blue) in male and female microglia at 2 and 16 DIV. Scale bar 150 µm. (b) Quantification of % of β‐gal positive cells per field. ^+^ *p* < .05 two‐way ANOVA effect of time. (c–e) miRNA expression of miRNA‐124a, miRNA‐146a, and miRNA‐155, measured as %RQ. (f–k) mRNA expression of Beclin‐1 (f), Toll‐like receptor (TLR)2 (g), TLR4 (h), interleukin (IL)‐1β (i), IL‐6 (j), and tumor necrosis factor α (TNF‐α; k), measured as %RQ. ^+++^ *p* < .001 effect of time, ^###^ *p* < .001 effect of time only in female microglia, measured by two‐way ANOVA followed by Bonferroni post hoc test. Dots show mean ± *SEM*. Blue: male; Purple: female

We first determined β‐galactosidase activity, which is increased in the senescence phenotype, at 2 and 16 days in vitro (DIV) in male and female microglial cells. There was a time‐dependent increase in the senescent phenotype regardless of the sex (Figure 2a,b). We also found decreased miRNA‐124a, miRNA‐146a, and miRNA‐155 expression, a characteristic of aged microglia, at 16 DIV in both sexes (Figure 2c‐e).

We next evaluated the mRNA expression of other senescence markers such as Beclin‐1, which plays a central role in autophagosome formation, TLR2 and TLR4, which are associated with microglia activation, and interleukin (IL)‐1β, IL‐6, and tumor necrosis factor‐α (TNF‐α), whose expression by microglia has been shown to be altered with physiological aging and in disease (Caldeira et al., 2014; Koellhoffer et al., 2017). We found decreased Beclin‐1, TLR2, and TLR4 and increased IL‐1β mRNA expression with time, in both male and female microglia (Figure 2f‐i). IL‐6 mRNA expression only decreased over time on female microglia (Figure 2j). In contrast, TNF‐α expression remained unaffected in both sexes (Figure 2k).

These results show that both male and female microglia acquire a senescent phenotype when kept in culture over 16 DIV, being the effect more pronounced in male than in female cells.

### 2.3 IFN‐γ induces a sex‐specific inflammatory response in primary microglia that is altered in the in vitro aging model

We next stimulated microglial cells at 2 and 16 DIV, using IFN‐γ as a pro‐inflammatory stimulus. The levels of this cytokine are increased in the aged brain, and converging evidences point to its involvement in different mechanisms of aging (Monteiro, Roque, Marques, Correia‐Neves, & Cerqueira, 2017).

**Figure 3:**
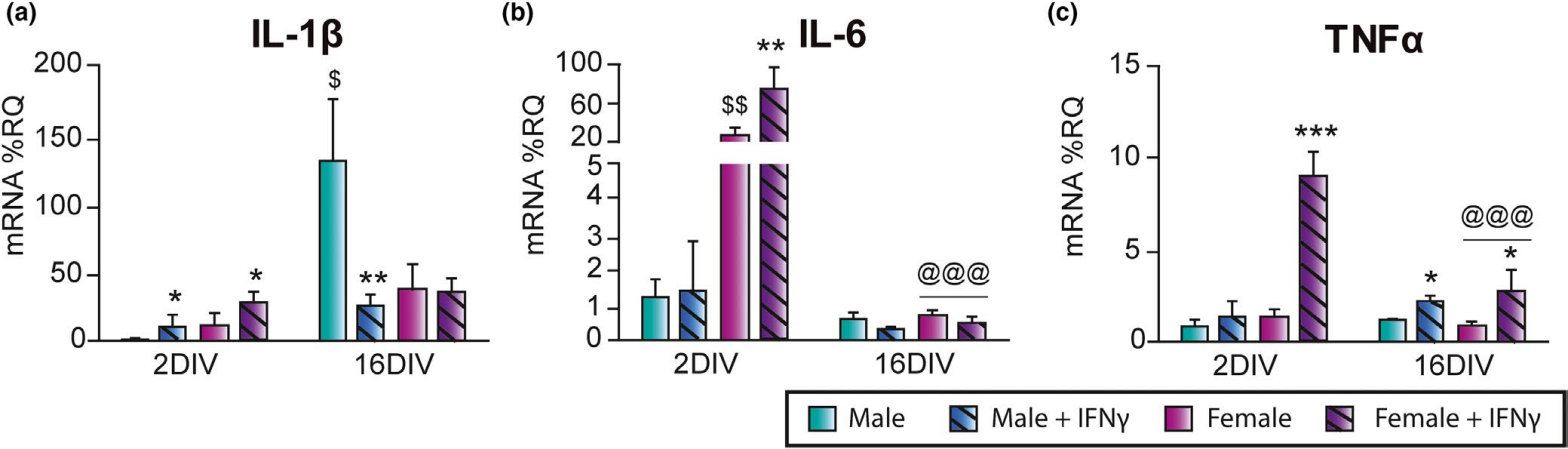
Evolution of the inflammatory profile of male and female microglia in an aging model in vitro. mRNA expression of interleukin (IL)‐1β (a), IL‐6 (b), and tumor necrosis factor α (TNF‐α; c) in microglial cells. *^@@@^p* < .001 effect of time on IFN‐γ‐induced mRNA expression by two‐way ANOVA followed by Bonferroni post hoc test; ^$^ *p* < .05, ^$$^ *p* < .01 sex differences; *p < .05, **p < .01, ***p < .001 effect of IFN‐γ treatment measured by one‐way ANOVA followed by Tukey's post hoc test. Dots show mean ± *SEM*. Blue: male; Purple: female; Dark and striped: IFN‐γ treatment

As observed in the previous experiment, female microglia showed increased basal levels of IL‐6 mRNA compared to male microglia at 2 DIV. However, by 16 DIV the basal mRNA levels of IL‐6 were reduced and reached male values (Figure 3b). In contrast, the basal levels of IL‐1β mRNA expression were increased in male microglia by 16 DIV over basal female levels (Figure 3a).

The effect of IFN‐γ was also different in male and female cells. Thus, IFN‐γ increased IL‐1β, IL‐6, and TNF‐α mRNA expression in female microglia at 2 DIV (Figure 3a‐c). In contrast, at 2 DIV, only the mRNA levels of IL‐1β were increased in male cells after the treatment with IFN‐γ; the mRNA levels of IL‐6 and TNF‐α remained unaffected (Figure 3a‐c). By 16 DIV, the effect of IFN‐γ on IL‐1β and IL‐6 mRNA levels in female microglia disappeared and the effect on TNF‐α mRNA expression was significantly reduced (Figure 3a‐c). In male cells at 16 DIV, IFN‐γ treatment reduced the mRNA levels of IL‐1β, in contrast to that observed in female microglia. However, IFN‐γ treatment increased the mRNA levels of TNF‐α in male cells at 16 DIV, as observed in females (Figure 3a‐c).

These results show that microglia derived from male or female brains show different inflammatory patterns, both under basal conditions and in response to IFN‐γ in the aging model in vitro.

### 2.4 Sex differences in microglia motility disappear after 16 DIV

In a healthy brain, microglial cells constantly monitor their immediate surroundings by extension and retraction of their motile processes, allowing homeostasis maintenance and fine‐tuning of neuronal activity. These cells also have the potential to move their soma, which allows a fast response for many pathophysiological processes (Kettenmann et al., 2011; Nimmerjahn, Kirchhoff, & Helmchen, 2005). Recent studies report that aged microglia show impaired migration and decreased motility and are unable to respond to several chemotactic stimuli in mice (Damani et al., 2011; Hefendehl et al., 2014). To test microglia motility in our aging model in vitro, we first analyzed the mRNA expression of migration‐related genes.

**Figure 4:**
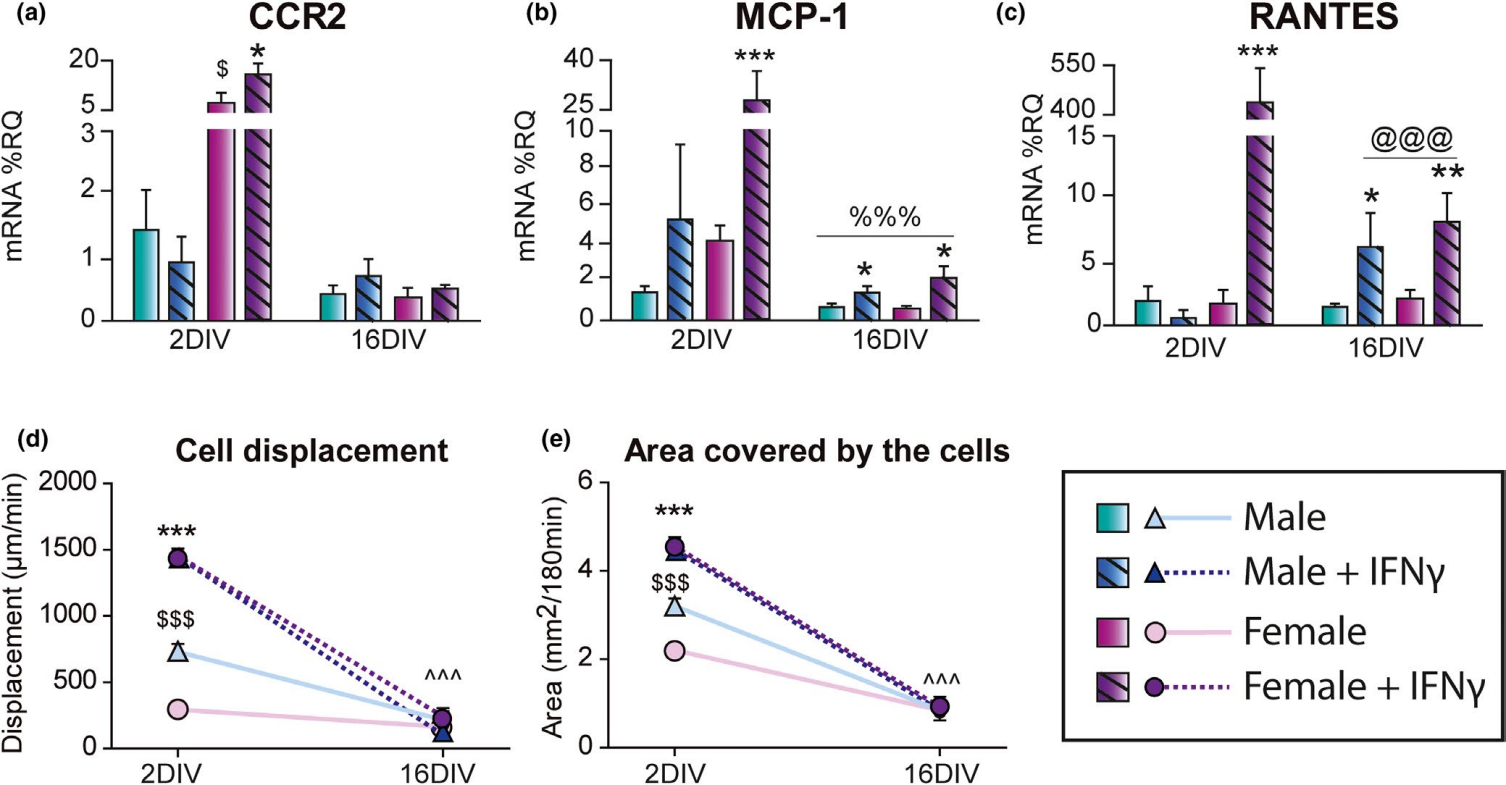
Evolution of migration capacity of microglia in an aging model in vitro. mRNA expression of monocyte chemotactic protein 1 (MCP‐1) receptor (CCR2; a), MCP‐1 (b), and regulated on activation, normal T cell expressed and secreted (RANTES; c), measured as %RQ, at 2 and 16 DIV. (d) Microglia displacement (area in µm^2^ a single cell moves around in 1 min). (e) Area covered by microglia (total area covered by a single cell in 3 hr, expressed in µm^2^). ^%%%^ *p* < .001 effect of time and treatment, ^^^^^ *p* < .001 effect of time and sex, *^@@@^p* < .001 effect of time on IFN‐γ‐induced mRNA expression, ^$$$^ *p* < .001 effect of sex measured by two‐way ANOVA followed by Bonferroni post hoc test; *p < .05, ***p < .001 effect of IFN‐γ treatment measured by one‐way ANOVA followed by Tukey's post hoc test. Dots show mean ± *SEM*. Blue: male; Purple: female; Dark and striped: IFN‐γ treatment

Results showed an effect of time and treatment in the mRNA expression of monocyte chemotactic protein 1 (MCP‐1) receptor (CCR2; Figure 4a), MCP‐1 (Figure 4b), and regulated on activation, normal T cell expressed and secreted (RANTES; Figure 4c). IFN‐γ treatment increased the mRNA levels of CCR2, MCP‐1, and RANTES (Figure 4a‐c) only in female microglia at 2 DIV and increased MCP‐1 and RANTES mRNA expression in male and female cells at 16 DIV.

Time‐lapse analysis of microglia showed that basal displacement and area covered by male microglia were higher than those of female microglia at 2 DIV (Figure 4d,e). IFN‐γ treatment increased motility of microglial cells regardless of the sex. Both parameters were decreased by time in male and female microglia, and the IFN‐γ‐induced increase in displacement and the area covered by the cells found at 2 DIV disappeared at 16 DIV (Figure 4d, e).

Therefore, microglia aged in vitro display a limited motility regardless of the sex, hence losing their physiological sex differences, and also lose the ability to respond to IFN‐γ stimulation.

### 2.5 In vitro aging alters the expression of phagocytosis receptors in microglia

**Figure 5:**
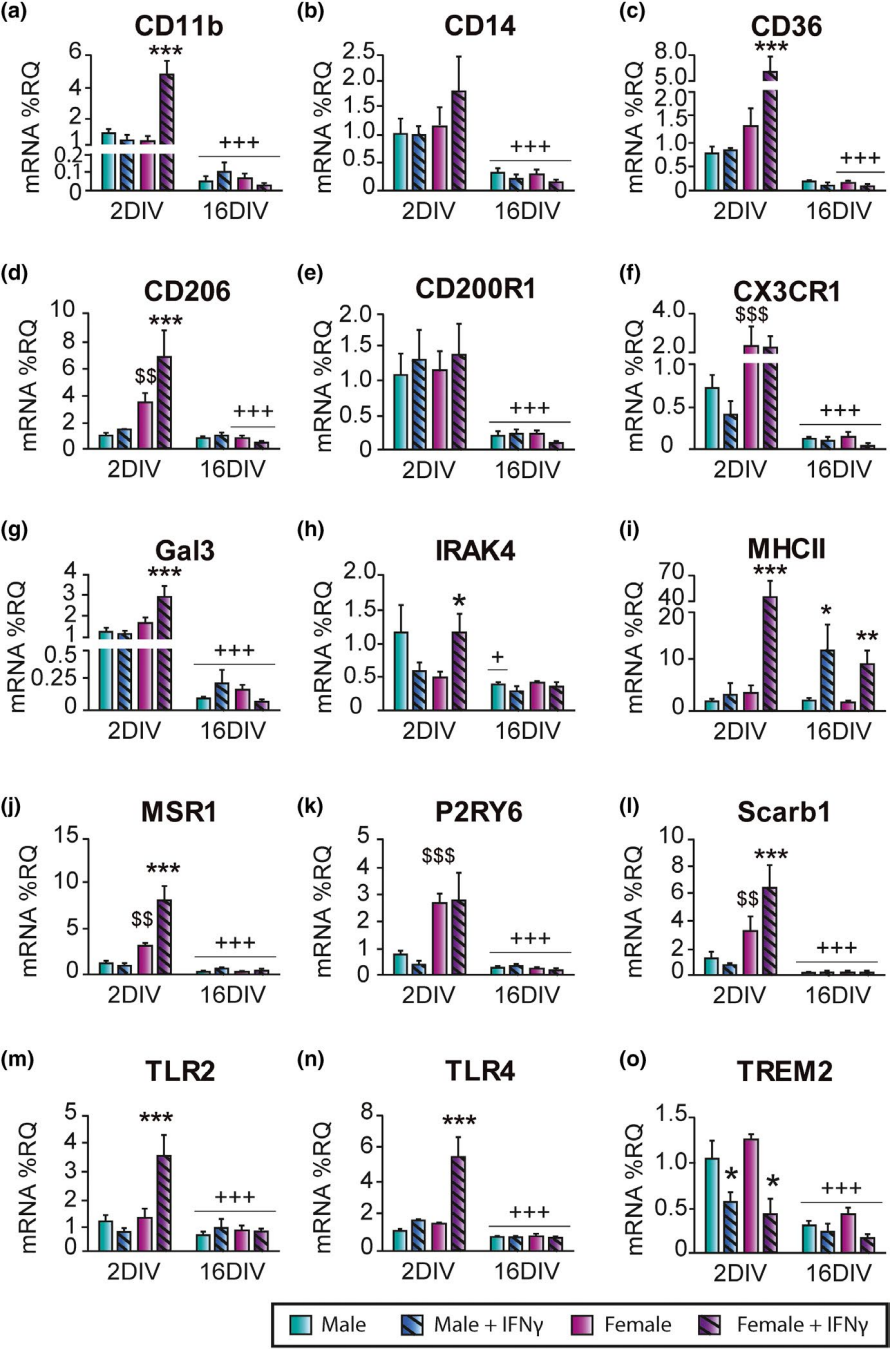
mRNA expression of phagocytosis‐related receptors in microglia at 2 and 16 days in vitro (DIV). mRNA expression of CD11b (a), CD14 (b), CD36 (c), mannose receptor (CD206; d), CD200R1 (e), C‐X3‐C motif receptor 1 (CX3CR1; f), galectin‐3 (Gal3; g), IL‐1 receptor‐associated kinase (IRAK)‐4 (h), MHCII (i), macrophage scavenger receptor 1 (MSR1; j), purine receptor P2Y6 (P2RY6; k), scavenger receptor class B member 1 (Scarb1; l), Toll‐like receptor (TLR)2 (m), TLR4 (n), and triggering receptor expressed on myeloid cells 2 (TREM2; o). ^+^ *p* < .05, ^+++^ *p* < .001 effect of time measured by two‐way ANOVA followed by Bonferroni post hoc test. *p < .05, **p < .01, ***p < .001 effect of IFN‐γ treatment, ^$$^ *p* < .01, ^$$$^ *p* < .001 sex differences measured by one‐way ANOVA followed by Tukey's post hoc test. Dots show mean ± *SEM*. Blue: male; Purple: female; Dark and striped: IFN‐γ treatment

As a first step to determine the effects of in vitro aging on microglia phagocytosis, we analyzed the mRNA expression of several genes implicated in this function at 2 and 16 DIV (Figure 5). At 2 DIV, female microglia showed higher basal mRNA levels of C‐X3‐C motif receptor 1 (CX3CR1; Figure 5f), mannose receptor (CD206; Figure 5d), macrophage scavenger receptor 1 (MSR1; Figure 5j), purine receptor P2Y6 (P2RY6; Figure 5k), and scavenger receptor class B member 1 (Scarb1; Figure 5l), than male microglia. IFN‐γ treatment increased the mRNA levels of CD11b, CD36, galectin‐3 (Gal3, also known as MAC‐2), CD206, MHCII, MSR1, Scarb1, TLR2, and TLR4 at 2 DIV, but only in female microglia (Figure 5a,c,g,i,j,l‐n).

Compared to 2 DIV, the mRNA expression levels of all genes tested decayed in microglia from both sexes at 16 DIV, except for CD36, which only decayed in female microglia, and IRAK4, which was downregulated in male microglia only. The effect of IFN‐γ treatment disappeared at 16 DIV in all cases except for MHCII (Figure 5). These results show that: (a) Female microglia at 2 DIV are more sensitive than male microglia to IFN‐γ treatment, regarding its effect on the mRNA expression of phagocytosis‐related genes, and (b) the aging process profoundly alters the expression of genes related to phagocytosis in microglia under basal conditions and after stimulation with IFN‐γ.

### 2.6 Microglia aged in vitro show similar alterations in phagocytosis activity as microglia purified from aged brains

To investigate whether microglia phagocytosis was affected by sex in our aging model in vitro, we performed the same engulfment assays we had previously tested in microglia isolated from adult and aged male and female brains, using IFN‐γ as a pro‐phagocytic stimulus (Yanguas‐Casás et al., 2018).

**Figure 6:**
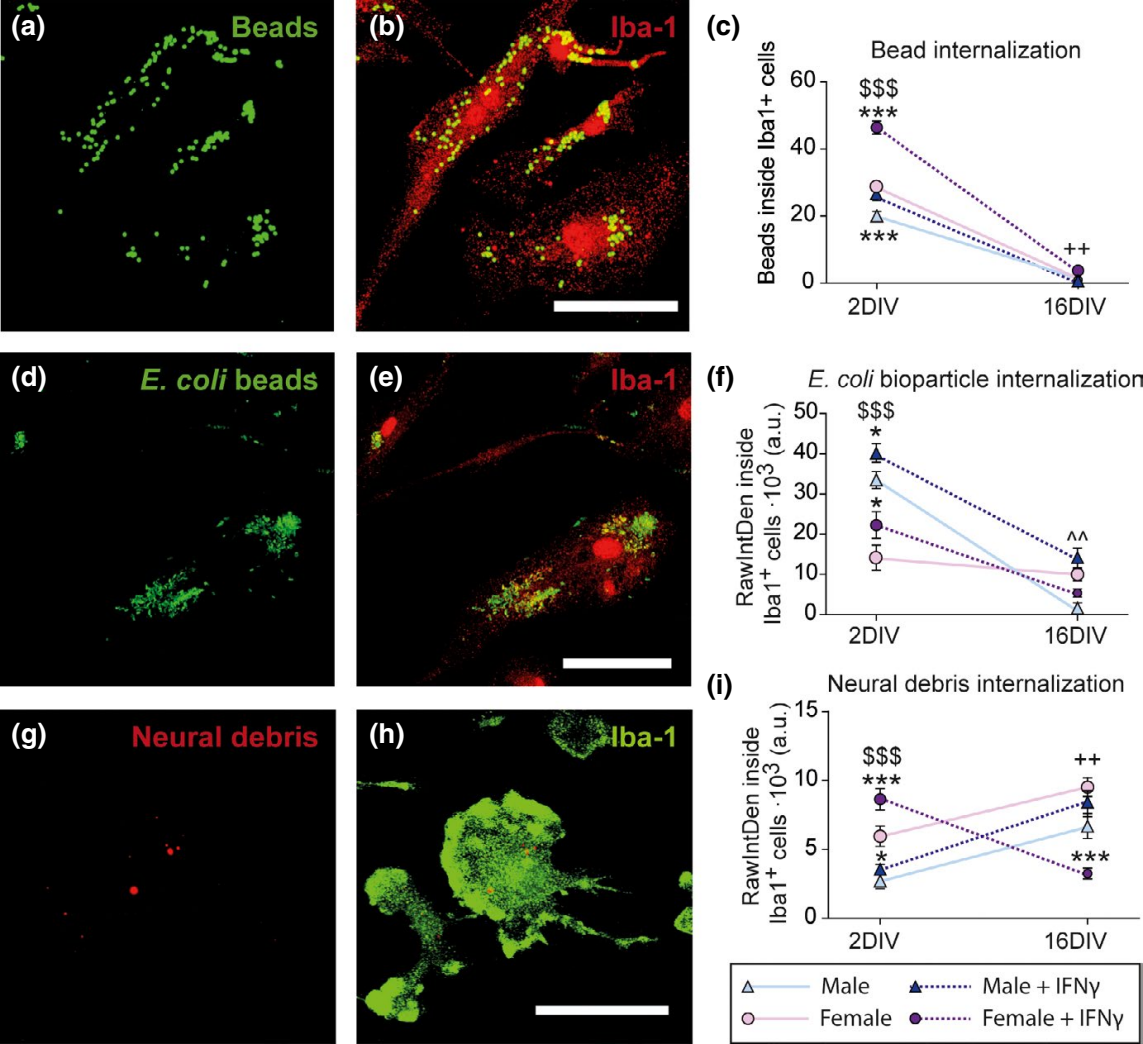
Microglia phagocytic capacity in an aging model in vitro. Representative images of microglia: nonspecific bead intake (a, b), pathogen‐specific (d, e), and neural debris (g, h) phagocytosis. (c, f, i) Amount of internalized particles per cell. ^++^ *p* < .01 effect of time in male and female microglia measured by two‐way ANOVA followed by Bonferroni post hoc test; ^^^^ *p < *.*01* effect of time in male microglia measured by two‐way ANOVA followed by Bonferroni post hoc test ^$$$^ *p* < .001 sex differences, *p < .05, ***p < .001 effect of IFN‐γ treatment measured by one‐way ANOVA followed by Tukey's post hoc test. Dots show mean ± *SEM*. Blue: male; Purple: female; Dark: IFN‐γ treatment

Male and female cells isolated from newborn brains showed different internalization patterns under basal conditions. Female microglia showed a higher basal internalization of fluorescent beads (Figure 6c) and neural debris (Figure 6i), while male microglia showed enhanced internalization of *E. coli* bioparticles (Figure 6f). The aging process in vitro imitated the effect of natural in vivo aging on microglia phagocytosis. Thus, both male and female microglia increased the internalization of neural debris with in vitro aging (Figure 6i), as it was observed in the comparison between microglia isolated from adult and aged brains. However, cells obtained from newborns showed an increased phagocytic activity in comparison with those isolated from adult and aged animals (Figure 1), in agreement with the fact that perinatal microglia present enhanced basal phagocytosis compared to adult microglia (Galloway et al., 2019; Lenz & Nelson, 2018).

IFN‐γ treatment increased nonspecific, pathogen‐specific, and neural debris intake in both male and female microglia at 2 DIV (Figure 6c,f,i). However, after IFN‐γ stimulation female microglia showed a much higher increase in the internalization of fluorescent beads and neural debris than male microglia at 2 DIV (Figure 6c). This sex difference was lost in aged (16 DIV) microglia, in which the increase in bead and neural debris phagocytosis upon IFN‐γ stimulation was not observed, neither in males nor in females (Figure 6c,i). Indeed, neural debris phagocytosis was even decreased upon IFN‐γ stimulation in female microglia at 16 DIV (Figure 6i). Thus, as it was observed in the comparison of microglia isolated from adult and aged brains, in vitro aging also affects microglia phagocytosis in response to an inflammatory challenge in a sex‐dependent fashion.

## 3 DISCUSSION

Our present findings, showing sex differences in the basal phagocytic activity of aging microglia and in their response to inflammatory stimuli, extend the results of previous studies that have characterized physiological and pathological sex differences in microglia during development and in adult life (Bordeleau, Carrier, Luheshi, & Tremblay, 2019; Rahimian, Cordeau, & Kriz, 2019). While those studies suggest that microglia are involved in the generation of sex differences in neurodevelopmental and psychiatric disorders and in the neurodegenerative response after acute traumatic brain injury or stroke in young animals, our findings have implications for the possible role of microglia in the generation of sex differences in the response of the aging brain to neurodegenerative conditions.

Reproducing microglia aging in vitro using long‐term murine cultures provides a useful tool to study senescent microglia, given the limitations of isolating degenerating microglia from the aged brains for experimentation, as only the more resistant microglia will survive the isolation procedure, while susceptible microglia are lost in this process (Njie et al., 2012). Microglia isolated from newborn and adult brains maintain sex‐specific features when in culture, such as postnatal sex differences in the phagocytic and migratory activity (Villa et al., 2018; Yanguas‐Casás et al., 2018). Our findings also indicate that microglia derived from newborn male or female mice brains acquire a similar phenotype when cultured during 16 DIV. This phenotype is characterized by increased β‐galactosidase activity, decreased expression of specific miRNAs and mRNA levels of Beclin‐1, TLR2, and TLR4, and reduced motility and mRNA expression of motility‐related genes (Caldeira et al., 2014; Sieber, Claus, Witte, & Frahm, 2011). Furthermore, microglia maintained for 16 DIV show an altered inflammatory response when exposed to IFN‐γ stimulation.

Although all these modifications are compatible with the phenotype of senescent microglia (Caldeira et al., 2014; Scheiblich, Trombly, Ramirez, & Heneka, 2020; Sieber et al., 2011), additional methods and molecular markers would be necessary to confirm cellular senescence in 16 DIV microglia cultures. Moreover, it is important to note that the molecular mechanisms that determine aging process of microglia in vitro may differ from those that cause microglia dysfunction in the aged brain (Stojiljkovic et al., 2019). For instance, the expression of Gal3 and MHCII in phagocytic cells has been shown to increase with age (Shobin et al., 2017). Here, we find that the mRNA expression of Gal3 decays in the aged phenotype of microglia, while MHCII mRNA expression levels remained unaffected. The expression of these markers is also linked to microglia priming (Holtman et al., 2015); therefore, these differences may be due to a different priming state of microglial cells aged in vitro or to the infiltration of macrophages and the expression of these markers by other cell types besides microglia in the aged brain. However, with independence of the differences in the triggering mechanisms, the observed functional characteristics of microglia aged in vitro are reminiscent of the functional changes that occur in microglia in vivo with the aging process, such as impaired inflammatory response (Scheiblich et al., 2020), decreased process motility, soma movement, and cellular migration and recruitment in the injured tissue compared to young microglia (Damani et al., 2011; Hefendehl et al., 2014).

In this study, our principal aim was to analyze the influence of aging and sex on microglia phagocytosis, a functional response of these cells that are involved in the regulation of brain tissue homeostasis under physiological and pathological conditions. Our findings indicate that donor's age had significant effects on basal sex differences in microglia phagocytosis in vitro. Thus, microglia isolated from newborn female brains showed a higher basal internalization of fluorescent beads and neural debris than male microglia. In contrast, male microglia from newborn animals showed higher internalization of *E. coli* bioparticles than female microglia. These sex differences disappeared in microglia isolated from adult (5 months) brains, which showed a similar basal internalization of fluorescent beads, *E. coli* bioparticles, and neural debris in both sexes.

Our results are in agreement with previous findings of sex differences in the activity of phagocytosis of developing microglia in vitro and in vivo (Weinhard et al., 2018; Yanguas‐Casás et al., 2018) and suggest that there are transient functional sex differences in basal microglia phagocytosis during postnatal development. During this period, male microglia showed preferentially pathogen‐specific phagocytosis, at least of gram‐negative bacteria substrate, while female microglia had enhanced nonspecific and neural debris phagocytosis. These differences, which may represent a sex‐dependent priority to trigger microbial pattern recognition‐mediated phagocytosis in developing male microglia or cellular debris clearance, induced phagocytosis in developing female microglia.

The results of the quantification of mRNA phagocytosis‐related genes are compatible with the functional sex differences detected in the phagocytosis assays. Thus, higher IRAK4 mRNA levels in microglia isolated from newborn male animals compared to female microglia are consistent with a higher capacity of male cells to internalize *E. coli*‐coated bioparticles, since IRAK4 mediates upregulation of scavenger receptors (Doyle et al., 2004) by TLR2 and TLR4. These receptors can recognize both microbial patterns and danger‐associated molecular patterns and mediate brain injury‐induced inflammation and microglia phagocytosis under pathological conditions (Fiebich, Batista, Saliba, Yousif, & de Oliveira, 2018). In addition, higher mRNA levels of CD206, MSR1, and Scarb1 in microglia isolated from newborn female brains are compatible with the increased basal nonspecific and neural debris phagocytosis of these cells, given that these genes encode for scavenger receptors that are important in the innate host response to bacterial and fungal pathogens (Husemann, Loike, Anankov, Febbraio, & Silverstein, 2002). However, in the absence of protein data these molecular interpretations remain speculative. Nevertheless, with independence of the possible molecular mechanisms, the observed functional differences in phagocytosis may have important consequences for the generation of sex differences in brain structure and adult brain function (Bordeleau et al., 2019; VanRyzin, Pickett, & McCarthy, 2018) and in the long‐term psychiatric consequences of perinatal infections (Ardalan et al., 2019). Further studies are still necessary to determine the mechanisms that generate the observed sex differences in the activity of phagocytosis of microglia, which may involve the perinatal sex hormone environment together with the cellular actions of sex chromosome genes (Loke, Harley, & Lee, 2015; VanRyzin et al., 2018).

In spite of the fact that basal sex differences in newborn microglia phagocytosis disappeared in adult microglia, cells from both ages responded to IFN‐γ stimulation by increasing nonspecific and pathogen‐specific phagocytic activity. However, an important finding in our study is that aging impaired the increase in nonspecific phagocytosis and reduced the increase in pathogen‐specific phagocytosis upon IFN‐γ stimulation in microglia of both sexes. The impaired effect of IFN‐γ on the stimulation of nonspecific and pathogen‐specific phagocytosis in aged microglia is consistent with the observed alterations in the mRNA expression of different molecules with in vitro aging. These include CD11b, CD14, CD36, CD206, CD200R1, CX3CR1, Gal3, MSR1, P2RY6, Scarb1, TLR2, TLR4, and TREM2, which are involved in different steps of microglia activation and the regulation of phagocytosis (Husemann et al., 2002; Neumann, Kotter, & Franklin, 2009).

One of the most relevant results of this study is that, while nonspecific and pathogen‐specific phagocytosis decay during the acquisition of the senescent phenotype, in agreement with the reported decrease in phagocytosis activity of aged microglia (Damani et al., 2011; Koellhoffer et al., 2017), neural debris phagocytosis increases with aging in male and female microglia. This effect was detected both in microglia isolated from aged brains and in microglia aged in vitro. We may speculate that the increase in neural debris uptake in aged microglia is more likely mediated through the deregulation of CX3CL1‐CX3CR1 and CD200‐CD200R1 axes, which have been correlated with microglia activation and exacerbated phagocytic responses (Oria et al., 2018; Raoul et al., 2010), rather than by a selective surface expression of debris clearance receptors in microglial cells, as mRNA expression of these receptors decays with time. However, further studies are necessary to unveil the precise mechanism and determine the functional consequences of this increase in phagocytosis activity by aging microglia. Nevertheless, as neuronal vulnerability increases with age, increased phagocytosis of neural debris might contribute to maintain homeostasis in the aged brain. Indeed, clearance of cellular debris from the parenchyma is essential to avoid further degeneration and exacerbated inflammatory responses in the brain under pathological conditions (Neumann et al., 2009). Alternatively, it may be hypothesized that the increased phagocytosis activity of neural debris by aged microglia could be part of the cell priming process associated with microglia senescence and may, therefore, contribute to brain deterioration with aging.

Another important observation in our study is that the phagocytosis of neural debris was impaired only in aged female microglia under inflammatory conditions elicited by IFN‐γ stimulation. This effect of aging may be associated with the marked decrease in P2RY6 mRNA expression detected in aged female microglia, since P2RY6 mediates UDP‐evoked phagocytosis of debris from damaged neurons (Koizumi et al., 2007). We can only speculate on the possible consequences of the impaired phagocytosis of neural debris by aging female microglia under inflammatory conditions. However, considering that brain aging is associated with increased neuroinflammation, our finding may implicate a decreased efficiency of microglia to maintain homeostasis in the aged female brain compared to the aged male brain or, on the contrary, a better control of the inflammatory stimulation of microglia phagocytosis by female cells to avoid further damage. In either case, the different phagocytic response of male and female microglia to inflammation may contribute to the generation of the well‐characterized sex differences in the incidence of neurodegenerative diseases with aging (Loke et al., 2015; The Lancet, 2019). Even though IFN‐γ is a factor aged microglia are physiologically exposed to (Monteiro et al., 2017), we cannot rule out that other inflammatory stimuli in vivo may elicit a different response or affect the one we are describing.

Finally, it should be emphasized that the sex‐specific alterations in the phagocytosis activity of aged microglia are associated with modifications in the inflammatory response that are also different between male and female cells and occur in parallel with impaired cell motility, a characteristic of microglia in several neurological diseases (O'Connor, Borsig, & Heikenwalder, 2015; Raoul et al., 2010). The combination of all these circumstances most probably will magnify the consequences of sex dimorphic microglia changes in the aged brain.

## 4 EXPERIMENTAL PROCEDURES

### 4.1 Animals

Postnatal P0‐P2 CD1 male and female mouse pups and C57BL/6 male mice were raised in our in‐house colony (Instituto Cajal, CSIC), and 8‐week‐old C57BL/6 female mice were obtained from Envigo. In all cases, mice were maintained under standard conditions (22°C with 50% ± 10% relative humidity and 12‐hr light/dark cycle) until the experimental procedures. Animal handling and care were performed in compliance with European Union guidelines 2010/63/EU and Spanish regulations (R.D. 53/2013) regarding the use and care of laboratory animals, and all protocols were approved by our local Animal Care and Ethics Committee (Comité de Ética de Experimentación Animal del Instituto Cajal) and by the Madrid regional government (Consejería del Medio Ambiente y Territorio, Comunidad de Madrid. Ref. PROEX 134/17).

### 4.2 Reagents

DNase I, papain, and dispase II were purchased from Sigma‐Aldrich. IFN‐γ was purchased from PeproTech EC, Ltd. Roswell Park Memorial Institute medium 1640 (RPMI), Dulbecco's modified Eagle's medium (DMEM), heat‐inactivated fetal bovine serum (FBS), and heat‐inactivated horse serum (HS) were purchased from Gibco BRL. Antibiotic–antimitotic, GlutaMAX^TM^ was purchased from Thermo Fisher Scientific. Percoll was purchased from GE Healthcare.

### 4.3 Microglia purification from adult mouse brain

5‐ and 18‐month‐old C57BL/6 male and female mice (4–5 animals per experimental group) were anesthetized with pentobarbital (Dolethal, 50 mg/kg body weight, intraperitoneal) and perfused transcardially with 0.9% saline. Adult or aged microglia were isolated as previously described (Lee & Tansey, 2013). Brain tissue was minced and digested at 37°C for 30 min with gentle shaking in a buffer containing papain, dispase II, and DNase I followed by mechanical dissociation. After neutralization of the reaction, cells were centrifuged and filtered by a 40‐µm mesh. Microglia fraction was obtained in a 30%–70% SIP Percoll gradient by centrifugation of the cells at 500g and 18°C for 30 min, with no brake. After centrifugation, myelin was discarded and 6ml of the interphase was collected and mixed with 40 ml RPMI. The cells were centrifuged at 500g and 18°C for 7 min with regular brake, and the pellet was resuspended on 2ml RPMI. After counting the cells, they were centrifuged at 168g for 10 min and resuspended in 180μL MACS buffer. Afterward, 10 μl/10^7^ cells of CD11b microbeads were added to the mix. After washing, magnetically labeled cells were collected using MACS column system (Miltenyi Biotec).

### 4.4 Microglia cultures from newborn mouse brain

Primary cultures of microglial cells were obtained from newborn (P0) to 2‐day‐old (P2) CD1 mouse forebrains. Pups were sexed via measurement of anogenital distance, and a separate cohort of animals was used for each experiment. Homogenized forebrains from male or female pups were grown separately in DMEM supplemented with 10% FBS, 10% HS, and P/S (DMEM 10:10:1) in 75‐cm^2^ flasks, coated with poly‐l‐lysine (10 μg/ml) as described previously (Mecha et al., 2011). Briefly, after reaching confluence, cells were shaken at 230 rpm for 3 hr at 37°C. Detached cells were centrifuged at 168g for 10 min. To avoid the estrogenic effects of phenol red, purified microglia were plated in warm antibiotic‐ and phenol red‐free RPMI 1640 supplemented with 0.1% FBS. All the subsequent procedures were carried out using this medium.

### 4.5 In vitro aging model

An age‐like phenotype was induced in microglia cultures as previously described (Caldeira et al., 2014), with minor modifications. After microglia purification from newborn brains, the cells were seeded on 6‐well plates coated with poly‐l‐lysine (10 μg/ml) at a density of 100,000 cells/cm^2^ for PCR analysis, 25,000 cells/cm^2^ for senescence assays, or 50,000 cells/cm^2^ for phagocytosis assays. Cells were maintained for 2, 10, or 16 days at 37º C and 5% CO_2_ in RPMI medium containing 0.5% FBS. The cells were incubated for at least 12 hr in antibiotic‐free serum‐free RPMI prior to IFN‐γ (20 ng/ml) treatment. Senescence, gene expression, phagocytic capacity, and motility of the cells were evaluated at the three time points.

### 4.6 Cell senescence

Microglia senescence was evaluated using the Senescence Cells Histochemical Staining Kit (Sigma) according to the manufacturer's protocol. Microglia were seeded at a density of 21,000 cells/cm^2^ and kept at 37°C in fresh RPMI with 0.5% FBS and P/S for 2 or 16 days. At the selected times, cells were washed with PBS, fixed for 7 min at room temperature, and stained for 2 hr at 37°C. Images for quantification of β‐galactosidase (senescent)‐positive cells were acquired using a 10× lens by phase contrast in a Leica DMI6000 microscope.

### 4.7 RNA purification of microglial cells and qPCR

To study microglial mRNA expression by quantitative PCR, microglial cells were seeded at a density of 100,000 cells/cm^2^ and lysed 24h after treatment with IFN‐γ at each time point (2 or 16 DIV). Total RNA was extracted using an Illustra RNAspin Mini RNA Isolation Kit (GE Healthcare) to assess the mRNA expression levels of the set of genes listed in Table 1. First‐strand cDNA was synthesized from 0.75 µg RNA using M‐MLV reverse transcriptase (Promega) according to the manufacturer's protocol.

**Table 1:** Mouse primers for quantitative PCR

Gene	Accession #	Forward primer 5′‐3′	Reverse primer 5′‐3′	Amplicon size (bp)
Beclin‐1		CTGGACAAGCTCAAGAAAACCAA	GCAAGCGACCCAGTCTGAAA	100
CCR2		CTCTGCAAACAGTGCCCAGTT	AACCGAGACCTCTTGCTCCCC	93
CD11b (Itgam)		ATGGACGCTGATGGCAATACC	TCCCCATTCACGTCTCCCA	203
	206		
CD14		CGTTGACGAGGACCCTCAGA	GCAGTGGCCTTGTCAGGAA	100
CD36		AGGTGATGGGTCTTCACCAG	ATTTGTGGTTGGTTGCCAAGG	110
CD200R1		CATAGGATGCATTTGTCTTTTGAAA	GCTGCATTTCATCCTCCTCAATA	98
CD206 (Mannose Receptor)		GGTTGGATTGAGGCCTGAAA	AACGTCCCTTTGTTTTGAACATC	66
CX3CR1		TGTCCTTTCTCTTTGTGAACATGA	GGCGGCGGCCATCTT	56
Gal3 (MAC2)		CTAATCAGGTGAGCGGCACAG	TCCTTGAGGGTTTGGGTTTCC	102
IL‐1b		GGTGTGTGACGTTCCCATTA	CCGACAGCACGAGGCTTT	74
IL‐6		GAAACCGCTATGAAGTTCCTCTCTG	TGTTGGGAGTGGTATCCTCTGTGA	136
IRAK4		GGCAATCTGAAGTCCCCTCGT	TCTGACGTTCCTCGCTTCCT	104
MCP‐1		TGTTGGCTCAGCCAGATGCAGTTA	TACAGCTTCTTTGGGACACCTGCT	131
MHCII		TTCCAGCCCCCATGTCAG	ACAACCCCAGGGCACAGA	54
MSR1		GAGTGTAGGCGGATCAACCC	CACTGGCCTTGGTGGAAGAT	96
P2RY6		CCAGTGCCAGGTTCAGGGTGTA	GCGTCTACCGTGAGGATTTCA	159
RANTES		ATATGGCTCGGACACCACTC	CGAGTGACAAAGACGACTGC	126
SCARB1		GATGTCACACCTGTCCGCA	CCTGGCTCACAGGCCATTTA	138
TLR2		TGTCCGCAATCATAGTTTCTGATG	AGCAGAGAAGTGAAGCCCCT	145
TLR4		GGCTCCTGGCTAGGACTCTGA	TCTGATCCATGCATTGGTAGGT	114
TNF‐α		GAAAAGCAAGCAGCCAACCA	CGGATCATGCTTTCTGTGCTC	106
TREM2		GCACCTCCAGGAATCAAGAG	GGGTCCAGTGAGGATCTGAA	200
RPL13A		TACCAGAAAGTTTGCTTACCTGGG	TGCCTGTTTCCGTAACCTCAAG	151
RPS29		ACATGTTCAGCCCGTATTTGC	GCCGCGTCTGCTCCAA	56

Diluted cDNA was amplified by real‐time PCR in a 15 µl volume reaction in a 7500 Real‐Time PCR System (Applied Biosystems) with Power SYBR^®^ Green reagent (Applied Biosystems). Gene expression was determined with 7500 Software v2.0.4 using ROX as passive reference dye. A standard curve with varying dilutions of each sample mix was performed for each set of primers to ensure the presence of unique amplification products. cDNA amplification was done in conventional Applied Biosystems cycling parameters (40 cycles of changing temperatures, first at 95°C for 15 s and then 60°C for 1 min).

Cycle threshold (*C*_t_) values for all analyzed genes ranged between 23 and 31. Data were represented using the comparative *C*_t_ method, and for a valid ΔΔ*C*_t_ value, we verified that the efficiency of amplification of the target and the reference gene was approximately equal (the absolute value of the slope of Δ*C*_t_ versus log relative concentration should be between −0.1 and 0.1). The *C*_t_ was determined for each target gene in duplicate. Δ*C*_t_ was calculated by the difference between the *C*_t_ of each target gene and the *C*_t_ of an artificial BestKeeper reference gene based on the *C*_t_ values of two independent reference genes: RPL13A and RPS29 calculated using the BestKeeper^©^ software (http://gene‐quantification.com/bestkeeper.html), which helps determine stable housekeeping *genes*, differentially regulated target *genes,* and sample integrity.

### 4.8 MicroRNA purification

MicroRNAs were purified following manufacturer's protocol of Isolation Kit *mir*Vana^™^. After acid phenol:chloroform extraction, miRNAs were isolated after washing with different ethanol concentrations, and collected with RNase‐free water. Less than 5 ng/µl miRNA were used in the retrotranscription/amplification phase. Following TaqMan^®^ Advanced miRNA Assays manufacturer's protocol, Poly(A) tailing reaction, adaptor ligation, and reverse transcription (RT) were made prior to miR amplification. After this, we proceeded with polymerase chain reaction (PCR), using different TaqMan Advanced miRNA Assay (20X): 480902_mir (miRNA‐124a), 481546_mir (miRNA‐146a), and 481328_mir (miRNA‐155). We used 18S (Mm03928990_g1, Thermo Fisher) as a housekeeping gene. cDNA amplification by real‐time quantitative PCR was done in 20 µl volume reaction in a 7500 Real‐Time PCR System (Applied Biosystems) with TaqMan Fast Advanced Master Mix (Thermo Fisher). Gene expression was determined with 7500 Software v2.0.4 and represented using the comparative *C*_t_ (cycle threshold) method.

### 4.9 Neuron debris production and labeling

Neurons were obtained from the brains of mouse embryos as previously described (Hilgenberg & Smith, 2007). Mouse embryos were sexed prior to brain extraction, and brains were minced and digested with a trypsin solution at 37°C for 15 min, and after mechanical disaggregation, the cells were resuspended in neurobasal medium containing B27, ampicillin, and GlutaMAX^TM^. After pelleting the cells by centrifugation at 168g for 5 min, neurons were resuspended in sodium carbonate buffer (0.1 M NaHCO_3_‐Na_2_CO_3_, pH 9.3) for optimal labeling and sonicated in a bar sonicator. Neuron debris was labeled using Cy^TM^3 Mono‐Reactive Dye Pack (Amersham Biosciences) according to the manufacturer's specifications and kept stored at 4°C until use.

### 4.10 Phagocytosis assays

To determine the microglia phagocytosis, cells were seeded on 10‐mm‐diameter glass coverslips coated with poly‐l‐lysine 10 μg/ml for in vitro microglia cultures or 50 μg/ml for adult mouse brain‐derived microglia at a density of 50,000 or 25,000 cells/cm^2^, respectively. After 24‐hr incubation in serum‐free RPMI or IFN‐γ (20 ng/ml) treatment, the cells were washed twice with warm RPMI medium and the phagocytosis reagents were added for 1 hr. Fluorescent beads (0.5 µl/well; Fluoresbrite^®^ YG Carboxylate Microspheres 1.00 µm, Polysciences, Inc.), Cy^TM^3‐labeled neural debris (5:1 microglia:neuron debris ratio), or *E. coli* bioparticles (1 µl/well; pHrodo^TM^ Green *E. coli* BioParticles^TM^ Conjugate for Phagocytosis, Invitrogen^TM^, Thermo Fisher Scientific) were added in warm RPMI at the selected wells. Afterward, cells were washed twice with warm PBS and fixed with 4% paraformaldehyde. Microglial cells were stained with rabbit anti‐Iba1 antibody (Wako Pure Chemical Industries; 1:500 dilution) followed by incubation with a goat anti‐rabbit Alexa 594‐conjugated secondary antibody (1:1,000) or guinea pig anti‐Iba1 antibody (Synaptic Systems) followed by incubation with a goat anti‐guinea pig Alexa 488‐conjugated secondary antibody (1:1,000). After washing with PBS, glass coverslips were mounted on slides with VECTASHIELD Antifade Mounting Medium with DAPI (Vector Laboratories). The Z‐stack images were visualized on a Leica TCS‐SP5 confocal system. Bead intake was quantified as beads inside the cell, and neural debris and *E. coli* bioparticle phagocytosis were quantified by measuring the raw intensity density using the Fiji software and maintaining the same threshold restrictions for all the experimental conditions. We characterized microglia phagocytosis as the amount of internalized bioparticles per cell in actively engulfing cells. Percentages of phagocytic microglia in each condition can be found on Table S1. In all cases, quantifications were performed in 50 cells from 5 fields per experimental condition per experiment, with five independent experiments being performed.

### 4.11 Time‐lapse acquisition of microglia motility

To study microglial motility, male and female microglial cells were seeded at a density of 25,000 cells/cm^2^ in Multiwell 6‐well plastic plates (FALCON, Corning Incorporated—Life Sciences) coated with poly‐l‐lysine (10 µg/ml).

Microglia motility was analyzed 2 or 16 days after microglia seeding, in cells obtained from the same cell culture. Phase‐contrast images of three fields per well were acquired every 2 min for 3 hr with a 203 0.70 DRY Leica DMI6000B lens in a time‐lapse Leica AF 6500–7000 microscope and analyzed using Fiji software. Microglia motility was evaluated using two parameters: total area covered by the cells during the acquisition (expressed as the area in mm^2^ covered by a single cell during a 3‐hr period) and the wandering of the cells (displacement, measured as the area in µm^2^ a single cell moves around in 1 min). Changes in cell shape and brightness did not allow a reliable automatic tracking, and therefore, this possibility was discarded. To determine the total area covered by each microglial cell, acquired images were stabilized and cropped, and the series of images was stacked with the StackReg plug‐in. The area covered by each cell was measured in the projection in µm^2^. Total path distance wandered by each cell was determined by tracking the trajectory of each cell body during the acquisition period with the Manual Tracking plug‐in.

### 4.12 Statistical analysis

The data in bar graphs are expressed as the mean ± *SEM*, and the data in scattered plots are expressed as the median ± range of a representative replicate, being the cells from the same replicate at the three time points analyzed when in vitro. GraphPad Prism software version 5.0 for Windows and SPSS 22 software (IBM Corporation) was used for the statistical analysis. Normality of the data was assessed with the Kolmogorov–Smirnov test, to satisfy the assumption of normality for the analysis of variance (ANOVA). Whenever normality was not achieved, statistical significance was determined with nonparametric tests (Kruskal–Wallis and post hoc pairwise comparisons with Mann–Whitney *U* test). One‐way ANOVA was used for comparison of multiple samples, followed by a Tukey's post hoc test to determine the statistical significance. Interactions between sex and treatment, sex and time, and time and treatment were determined using the two‐way ANOVA interaction model, with post hoc Bonferroni's comparisons. Statistical significance was set at *p* < .05 in all cases.

## CONFLICT OF INTEREST

The authors of the manuscript declare no conflict of interest. They certify that they have no affiliations with or involvement in any organization or entity with any financial interest (such as honoraria; educational grants; participation in speakers’ bureaus; membership, employment, consultancies, stock ownership, or other equity interest; and expert testimony or patent‐licensing arrangements), or nonfinancial interest (such as personal or professional relationships, affiliations, knowledge, or beliefs) in the subject matter or materials discussed in this manuscript.

## AUTHOR CONTRIBUTIONS

NYC conceived and designed the study, and analyzed and interpreted the data. ACC and NYC conducted the experimental procedures. LMGS and MAA advised on the experimental design and contributed materials and animals. All authors contributed to manuscript writing and revision and approved the final version.

## Supporting information

Table S1

## Data Availability

The data that support the findings of this study are available from the corresponding author upon reasonable request.

## References

[acel13182-bib-0001] Ardalan, M. , Chumak, T. , Vexler, Z. , & Mallard, C. (2019). Sex‐dependent effects of perinatal inflammation on the brain: Implication for neuro‐psychiatric disorders. International Journal of Molecular Sciences, 20(9), 2270 10.3390/ijms20092270 PMC6539135 31071949

[acel13182-bib-0002] Bachiller, S. , Jimenez‐Ferrer, I. , Paulus, A. , Yang, Y. , Swanberg, M. , Deierborg, T. , & Boza‐Serrano, A. (2018). Microglia in neurological diseases: A road map to brain‐disease dependent‐inflammatory response. Frontiers in Cellular Neuroscience, 12, 488 10.3389/fncel.2018.00488 30618635 PMC6305407

[acel13182-bib-0003] Bordeleau, M. , Carrier, M. , Luheshi, G. N. , & Tremblay, M. E. (2019). Microglia along sex lines: From brain colonization, maturation and function, to implication in neurodevelopmental disorders. Seminars in Cell & Developmental Biology, 94, 152–163. 10.1016/j.semcdb.2019.06.001 31201858

[acel13182-bib-0004] Caldeira, C. , Oliveira, A. F. , Cunha, C. , Vaz, A. R. , Falcao, A. S. , Fernandes, A. , & Brites, D. (2014). Microglia change from a reactive to an age‐like phenotype with the time in culture. Frontiers in Cellular Neuroscience, 8, 152 10.3389/fncel.2014.00152 24917789 PMC4040822

[acel13182-bib-0005] Damani, M. R. , Zhao, L. , Fontainhas, A. M. , Amaral, J. , Fariss, R. N. , & Wong, W. T. (2011). Age‐related alterations in the dynamic behavior of microglia. Aging Cell, 10(2), 263–276. 10.1111/j.1474-9726.2010.00660.x 21108733 PMC3056927

[acel13182-bib-0006] Doyle, S. E. , O'Connell, R. M. , Miranda, G. A. , Vaidya, S. A. , Chow, E. K. , Liu, P. T. , … Cheng, G. (2004). Toll‐like receptors induce a phagocytic gene program through p38. Journal of Experimental Medicine, 199(1), 81–90. 10.1084/jem.20031237 14699082 PMC1887723

[acel13182-bib-0007] Fiebich, B. L. , Batista, C. R. A. , Saliba, S. W. , Yousif, N. M. , & de Oliveira, A. C. P. (2018). Role of microglia TLRs in neurodegeneration. Frontiers in Cellular Neuroscience, 12, 329 10.3389/fncel.2018.00329 30333729 PMC6176466

[acel13182-bib-0008] Fu, R. , Shen, Q. , Xu, P. , Luo, J. J. , & Tang, Y. (2014). Phagocytosis of microglia in the central nervous system diseases. Molecular Neurobiology, 49(3), 1422–1434. 10.1007/s12035-013-8620-6 24395130 PMC4012154

[acel13182-bib-0009] Galloway, D. A. , Phillips, A. E. M. , Owen, D. R. J. , & Moore, C. S. (2019). Phagocytosis in the Brain: Homeostasis and Disease. Frontiers in Immunology, 10, 790 10.3389/fimmu.2019.00790 31040847 PMC6477030

[acel13182-bib-0010] Hanamsagar, R. , & Bilbo, S. D. (2016). Sex differences in neurodevelopmental and neurodegenerative disorders: Focus on microglial function and neuroinflammation during development. Journal of Steroid Biochemistry and Molecular Biology, 160, 127–133. 10.1016/j.jsbmb.2015.09.039 26435451 PMC4829467

[acel13182-bib-0011] Hefendehl, J. K. , Neher, J. J. , Suhs, R. B. , Kohsaka, S. , Skodras, A. , & Jucker, M. (2014). Homeostatic and injury‐induced microglia behavior in the aging brain. Aging Cell, 13(1), 60–69. 10.1111/acel.12149 23953759 PMC4326865

[acel13182-bib-0012] Hilgenberg, L. G. , & Smith, M. A. (2007). Preparation of dissociated mouse cortical neuron cultures. Journal of Visualized Experiments (10), 562 10.3791/562 18989405 PMC2557074

[acel13182-bib-0013] Holtman, I. R. , Raj, D. D. , Miller, J. A. , Schaafsma, W. , Yin, Z. , Brouwer, N. , … Eggen, B. J. L. (2015). Induction of a common microglia gene expression signature by aging and neurodegenerative conditions: A co‐expression meta‐analysis. Acta Neuropathologica Communications, 3, 31 10.1186/s40478-015-0203-5 26001565 PMC4489356

[acel13182-bib-0014] Husemann, J. , Loike, J. D. , Anankov, R. , Febbraio, M. , & Silverstein, S. C. (2002). Scavenger receptors in neurobiology and neuropathology: Their role on microglia and other cells of the nervous system. Glia, 40(2), 195–205. 10.1002/glia.10148 12379907

[acel13182-bib-0015] Kettenmann, H. , Hanisch, U. K. , Noda, M. , & Verkhratsky, A. (2011). Physiology of microglia. Physiological Reviews, 91(2), 461–553. 10.1152/physrev.00011.2010 21527731

[acel13182-bib-0016] Koellhoffer, E. C. , McCullough, L. D. , & Ritzel, R. M. (2017). Old maids: Aging and its impact on microglia function. International Journal of Molecular Sciences, 18(4), 10.3390/ijms18040769 PMC5412353 28379162

[acel13182-bib-0017] Koizumi, S. , Shigemoto‐Mogami, Y. , Nasu‐Tada, K. , Shinozaki, Y. , Ohsawa, K. , Tsuda, M. , … Inoue, K. (2007). UDP acting at P2Y6 receptors is a mediator of microglial phagocytosis. Nature, 446(7139), 1091–1095. 10.1038/nature05704 17410128 PMC3464483

[acel13182-bib-0018] Lee, J. K. , & Tansey, M. G. (2013). Microglia isolation from adult mouse brain. Methods in Molecular Biology, 1041, 17–23. 10.1007/978-1-62703-520-0_3 23813365 PMC4145600

[acel13182-bib-0019] Lenz, K. M. , & Nelson, L. H. (2018). Microglia and beyond: Innate immune cells as regulators of brain development and behavioral function. Frontiers in Immunology, 9, 698 10.3389/fimmu.2018.00698 29706957 PMC5908908

[acel13182-bib-0020] Liang, S. , Domon, H. , Hosur, K. B. , Wang, M. , & Hajishengallis, G. (2009). Age‐related alterations in innate immune receptor expression and ability of macrophages to respond to pathogen challenge in vitro. Mechanisms of Ageing and Development, 130(8), 538–546. 10.1016/j.mad.2009.06.006 19559723 PMC2717634

[acel13182-bib-0021] Loke, H. , Harley, V. , & Lee, J. (2015). Biological factors underlying sex differences in neurological disorders. International Journal of Biochemistry & Cell Biology, 65, 139–150. 10.1016/j.biocel.2015.05.024 26028290

[acel13182-bib-0022] Mecha, M. , Iñigo, P. M. , Mestre, L. , Hernangómez, M. , Borrell, J. , & Guaza, C. (2011). An easy and fast way to obtain a high number of glial cells from rat cerebral tissue: A beginners approach. Protocol Exchange, 218, 10.1038/protex.2011.218

[acel13182-bib-0023] Monteiro, S. , Roque, S. , Marques, F. , Correia‐Neves, M. , & Cerqueira, J. J. (2017). Brain interference: Revisiting the role of IFNgamma in the central nervous system. Progress in Neurobiology, 156, 149–163. 10.1016/j.pneurobio.2017.05.003 28528956

[acel13182-bib-0024] Neumann, H. , Kotter, M. R. , & Franklin, R. J. (2009). Debris clearance by microglia: An essential link between degeneration and regeneration. Brain, 132(Pt 2), 288–295. 10.1093/brain/awn109 18567623 PMC2640215

[acel13182-bib-0025] Nimmerjahn, A. , Kirchhoff, F. , & Helmchen, F. (2005). Resting microglial cells are highly dynamic surveillants of brain parenchyma in vivo. Science, 308(5726), 1314–1318. 10.1126/science.1110647 15831717

[acel13182-bib-0026] Njie, E. G. , Boelen, E. , Stassen, F. R. , Steinbusch, H. W. , Borchelt, D. R. , & Streit, W. J. (2012). Ex vivo cultures of microglia from young and aged rodent brain reveal age‐related changes in microglial function. Neurobiology of Aging, 33(1), 195 e191–112 10.1016/j.neurobiolaging.2010.05.008 PMC4162517 20580465

[acel13182-bib-0027] O'Connor, T. , Borsig, L. , & Heikenwalder, M. (2015). CCL2‐CCR2 signaling in disease pathogenesis. Endocrine, Metabolic & Immune Disorders: Drug Targets, 15(2), 105–118. 10.2174/1871530315666150316120920 25772168

[acel13182-bib-0028] Oria, M. , Figueira, R. L. , Scorletti, F. , Sbragia, L. , Owens, K. , Li, Z. , … Peiro, J. L. (2018). CD200‐CD200R imbalance correlates with microglia and pro‐inflammatory activation in rat spinal cords exposed to amniotic fluid in retinoic acid‐induced spina bifida. Scientific Reports, 8(1), 10638 10.1038/s41598-018-28829-5 30006626 PMC6045622

[acel13182-bib-0029] Rahimian, R. , Cordeau, P. Jr , & Kriz, J. (2019). Brain response to injuries: When microglia go sexist. Neuroscience, 405, 14–23. 10.1016/j.neuroscience.2018.02.048 29526689

[acel13182-bib-0030] Ransohoff, R. M. , & Perry, V. H. (2009). Microglial physiology: Unique stimuli, specialized responses. Annual Review of Immunology, 27, 119–145. 10.1146/annurev.immunol.021908.132528 19302036

[acel13182-bib-0031] Raoul, W. , Auvynet, C. , Camelo, S. , Guillonneau, X. , Feumi, C. , Combadiere, C. , & Sennlaub, F. (2010). CCL2/CCR2 and CX3CL1/CX3CR1 chemokine axes and their possible involvement in age‐related macular degeneration. Journal of Neuroinflammation, 7, 87 10.1186/1742-2094-7-87 21126357 PMC3003653

[acel13182-bib-0032] Rawji, K. S. , Mishra, M. K. , Michaels, N. J. , Rivest, S. , Stys, P. K. , & Yong, V. W. (2016). Immunosenescence of microglia and macrophages: Impact on the ageing central nervous system. Brain, 139(Pt 3), 653–661. 10.1093/brain/awv395 26912633 PMC5839598

[acel13182-bib-0033] Scheiblich, H. , Trombly, M. , Ramirez, A. , & Heneka, M. T. (2020). Neuroimmune connections in aging and neurodegenerative diseases. Trends in Immunology, 41(4), 300–312. 10.1016/j.it.2020.02.002. 32147113

[acel13182-bib-0034] Shobin, E. , Bowley, M. P. , Estrada, L. I. , Heyworth, N. C. , Orczykowski, M. E. , Eldridge, S. A. , … Rosene, D. L. (2017). Microglia activation and phagocytosis: Relationship with aging and cognitive impairment in the rhesus monkey. Geroscience, 39(2), 199–220. 10.1007/s11357-017-9965-y 28238188 PMC5411373

[acel13182-bib-0035] Sieber, M. W. , Claus, R. A. , Witte, O. W. , & Frahm, C. (2011). Attenuated inflammatory response in aged mice brains following stroke. PLoS One, 6(10), e26288 10.1371/journal.pone.0026288 22028848 PMC3196544

[acel13182-bib-0036] Stojiljkovic, M. R. , Ain, Q. , Bondeva, T. , Heller, R. , Schmeer, C. , & Witte, O. W. (2019). Phenotypic and functional differences between senescent and aged murine microglia. Neurobiology of Aging, 74, 56–69. 10.1016/j.neurobiolaging.2018.10.007 30439594

[acel13182-bib-0037] Streit, W. J. , & Xue, Q. S. (2013). Microglial senescence. CNS & Neurological Disorders: Drug Targets, 12(6), 763–767. 24047521 10.2174/18715273113126660176

[acel13182-bib-0038] The Lancet, N (2019). A spotlight on sex differences in neurological disorders. The Lancet Neurology, 18(4), 319 10.1016/S1474-4422(19)30001-8 30878094

[acel13182-bib-0039] VanRyzin, J. W. , Pickett, L. A. , & McCarthy, M. M. (2018). Microglia: Driving critical periods and sexual differentiation of the brain. Developmental Neurobiology, 78(6), 580–592. 10.1002/dneu.22569 29243403 PMC5980665

[acel13182-bib-0040] Villa, A. , Gelosa, P. , Castiglioni, L. , Cimino, M. , Rizzi, N. , Pepe, G. , … Maggi, A. (2018). Sex‐Specific Features of Microglia from Adult Mice. Cell Reports, 23(12), 3501–3511. 10.1016/j.celrep.2018.05.048 29924994 PMC6024879

[acel13182-bib-0041] Weinhard, L. , Neniskyte, U. , Vadisiute, A. , di Bartolomei, G. , Aygun, N. , Riviere, L. , … Gross, C. (2018). Sexual dimorphism of microglia and synapses during mouse postnatal development. Developmental Neurobiology, 78(6), 618–626. 10.1002/dneu.22568 29239126 PMC6001780

[acel13182-bib-0042] Yanguas‐Casás, N. , Crespo‐Castrillo, A. , de Ceballos, M. L. , Chowen, J. A. , Azcoitia, I. , Arevalo, M. A. , & Garcia‐Segura, L. M. (2018). Sex differences in the phagocytic and migratory activity of microglia and their impairment by palmitic acid. Glia, 66(3), 522–537. 10.1002/glia.23263 29139169

