# Supplementary material for: Aging and sex: Impact on microglia phagocytosis
Source: Aging Cell. 2020 Jul 29;19(8):e13182. doi: 10.1111/acel.13182 (PMC7431836; doi:10.1111/acel.13182)
Supplement: Supplementary file 1 — Table S1 [file ACEL-19-e13182-s001.docx]

**Table 1. Percentage of phagocytic microglia.**

|  | Beads | | | | | *E. coli* bioparticles | | | | | Neural Debris | | | | |
| --- | --- | --- | --- | --- | --- | --- | --- | --- | --- | --- | --- | --- | --- | --- | --- |
| Microglia  (age) | Male | Male + IFN | Female | Female + IFN | Male | | Male + IFN | Female | Female + IFN | Male | | Male + IFN | Female | Female + IFN |  |
| P. 2DIV | 48.73  ± 6.75 | 54.00 ± 17.58 | 57.44 ± 13.31 | 57.53  ± 23.23 | 49.41 ± 19.08 | | 60.02 ± 28.23 | 30.47 ± 18.27 | 28.22 ± 7.504 | 17.38 ± 14.40 | | 45.30 ± 20.69 | 42.68 ± 7.487 | 59.23 ± 16.65 |  |
| P. 16DIV | 35.40 ± 19.79 | 44.20 ± 26.33 | 48.42 ±  24.5 | 66.83 ± 14.32 | 19.51 ± 6.303 | | 28.76 ± 9.119 | 42.07 ± 16.32 | 50.50 ± 6.851 | 54.86 ± 23.45 | | 44.09 ± 23.93 | 55.04 ± 15.04 | 55.59 ± 9.592 |  |
| Adult  (5 mo) | 70.75 ± 7.588 | 78.75 ± 10.31 | 72.88 ± 6.486 | 77.00 ± 10.42 | 91.00 ± 3.742 | | 95.00 ± 4.082 | 94.75 ± 2.500 | 93.25 ± 2.754 | 94.75 ± 6.076 | | 89.25 ± 7.676 | 96.25 ± 4.787 | 94.75 ±  4.5 |  |
| Old  (18 mo) | 75.24 ± 10.34 | 85.50 ± 10.34 | 95.25 ± 1.4161 | 75.48 ± 10.54 | 87.79 ± 0.972 | | 94.17 ± 10.07 | 94.96 ± 3.410 | 94.13 ± 4.211 | 100  ±  0.00 | | 100  ± 0.00 | 100  ±  0.00 | 100  ±  0.00 |  |

Values represent Mean ± SD of the percentage of phagocytic cells in five fields per condition in at least three independent replicates. P.: postnatal; DIV: days *in vitro*; mo: month old.
